# The Efficacy of Vaccination

**Published:** 1883-05

**Authors:** 


### THE EFFICACY OF VACCINATION.

MR. BUCHANAN, taking the metropolis alone as the field of his investigation, adduces evidence of a still more striking kind. During the fifty-two weeks which ended on the 29th of May, 1881, the deaths in London from smallpox were, of each age of the vaccinated class, including all ages, 90 per million ; while the deaths in the unvaccinated class were 3,350 per million. Under twenty years the deaths in the vaccinated class were 61 per million, against 4,520 in the unvaccinated, and under five years the deaths were 40.5 in the vaccinated class, as against 5,950 in the unvaccinated. The word vaccinated is used in its widest scope, to embrace even an alleged performance of the operation ; and Dr. Buchanan points out that the population of London, under ten years of age, was 916,784 on the census night of 1881. This population divides, in round numbers, into 55,090 unvaccinated and 861,000 vaccinated. The actual mortality from smallpox during 1881 among children under ten was 782 among the 55,000 who had not been vaccinated, and 125 among the 861,000 who had been vaccinated. Upon equal numbers of both classes, therefore, the mortality from smallpox among the unvaccinated was about a hundredfold that among the vaccinated ; and this degree of protection was given to children under ten by the current vaccination, good and bad, of the metropolis. If the vaccinated children had died of smallpox at the same rate as the unvaccinated, the mortality among them would have been, instead of 125, more than 12,000 ; and if the unvaccinated children had been preserved in the same proportion as the vaccinated, the mortality among them, instead of being 782, would have been only *nine*.

Dr. Buchanan purposely confines his observations to children under ten, because he does not wish to complicate the question by any consideration of the need for or the efficacy of revaccination at some stated period. He claims, and with good reason, that in the single year 1881 the existing vaccination law was instrumental, in London alone, in saving the lives of 12,000 children, who, but for its operation, or but for the practice which it enforces, would have died of smallpox. On the other side of the question, he finds that in the same year the deaths of twelve children were registered in London as having been due to cowpox or disease occurring after vaccination. Inquiry into the cases showed that the cause of death was, with hardly an exception, erysipelas, and that its dependence upon vaccination was at least doubtful ; but, taking things at the worst, we have a clear gain of 1,000 lives for each one that was sacrificed.—*London Times.*

---

When a swimmer gets chilled the blood ceases to circulate in the fingers, the finger nails become a deadly white color, the lips turn blue, and should he persist in staying in the water after these symptoms develop he is sure to have cramps. So long as the swimmer can discern spots on his finger nails he knows that his blood is in good order, and that he is safe and free from chills.

